# Real-world off-label apixaban dosing and clinical outcomes in patients with heart failure and end-stage kidney disease

**DOI:** 10.1097/MD.0000000000049075

**Published:** 2026-05-29

**Authors:** Sung Il Im, Su Hyun Bae, Soo Jin Kim, Bong Joon Kim, Jung Ho Heo, Ye Na Kim, Yeonsoon Jung, Hark Rim, Sung Pil Cho, Jung Hwan Park, Ho Sik Shin

**Affiliations:** a Division of Cardiology, Department of Medicine, Kosin University Gospel Hospital, Kosin University College of Medicine, Busan, Korea; b Renal Division, Department of Internal Medicine, Gospel Hospital, Kosin University College of Medicine, Busan, South Korea; c Transplantation Research Institute, Kosin University College of Medicine, Busan, South Korea; d MEZOO Co., Ltd., Wonju-si, Gangwon-do, Korea.

**Keywords:** Apixaban, atrial fibrillation, end stage kidney disease, heart failure, off-label dosing

## Abstract

Apixaban plays a crucial role in preventing cardioembolic events in patients with nonvalvular atrial fibrillation (NVAF). However, in clinical practice, physicians often adjust apixaban dosing based on kidney function, deviating from guideline-recommended dosing. This study investigated the effects of real-world, off-label apixaban dosing on long-term outcomes in patients with heart failure (HF), NVAF, and end-stage kidney disease (ESKD). We analyzed data from a HF registry of patients with NVAF and ESKD between 2018 and 2024. The inclusion criteria comprised all patients treated with apixaban. Patients were categorized according to the strength of the apixaban dose administered. Outcomes, including bleeding events, systemic thromboembolic events, and all-cause mortality, were compared. Among 480 patients, 265 (55.2%), with a mean age of 77.3 ± 10.1 years, received an off-label underdose of apixaban. Baseline characteristics, including CHA_2_DS_2_-VASc and HAS-BLED scores, were similar across groups. Over a median follow-up of 48 months, no significant differences in systemic thromboembolic events or mortality were observed between the off-label underdose group and those receiving standard doses or off-label overdoses (*P* = .705). Similarly, no significant differences were observed in bleeding risk (underdose vs standard, *P* = .600; overdose vs standard, *P* = .395; underdose vs overdose, *P* = .469). In multivariate analysis, the CHA_2_DS_2_-VASc score was an independent predictor of thromboembolic events (odds ratio 1.818, 95% confidence interval 1.081–3.058; *P* = .024). In patients with HF, NVAF, and ESKD, off-label apixaban dosing was not associated with an increased risk of systemic thromboembolic events, mortality, or bleeding, highlighting the potential for personalized apixaban dosing based on patient-specific factors and pharmacokinetics, particularly in Asian populations.

## 1. Introduction

End-stage kidney disease (ESKD; estimated glomerular filtration rate [eGFR] < 30 mL/min/1.73m^2^) is a global public health burden associated with high morbidity and mortality. In addition, cardiovascular (CV) disease is a major cause of death in patients with ESKD, both with and without hemodialysis (HD).^[1]^

The incidence of atrial fibrillation (AF) risk is higher (3 times) in patients with ESKD than in the general population.^[2,3]^ The thromboembolism risk associated with AF is also independently higher in patients with ESKD than in those without ESKD.^[4]^ The annual stroke incidence in patients with ESKD is 5 times higher, even with no risk factor (CHA_2_DS_2_-VASc score of 0).^[5]^ Stroke risk increases with advancing stages of kidney disease (KD), and ESKD patients who underwent HD are associated with risk of new-onset AF.^[6]^ Those ESKD patients with AF have increased stroke rates (1.6 time higher) than those without AF in the United States Renal Data System.^[7]^ ESKD patients with stroke have very desperate clinical outcomes including higher mortality rate (around 30% annually). AF itself can also increase the risk of early-stage KD processing to ESKD.^[8]^

Furthermore, these patients are prone to an increased bleeding risk, primarily due to factors such as uremia-induced platelet dysfunction, impaired interactions between platelets and the vessel wall, and the use of heparin during HD sessions.^[9]^ The estimated risk of major bleeding is at least 2-fold higher in patients with renal impairment and at least 3-fold higher in patients requiring dialysis compared with patients with normal renal function.^[6]^ Therefore, anticoagulant strategies must be carefully and intricately balanced. This situation is further complicated by a lack of clarity regarding the most appropriate anticoagulation approach for this specific patient subset.

The patients with both HF and KD are at increased risk of cardiovascular events and progression to ESKD, in spite of heart failure (HF) management advancement.^[10]^ Many patients with HF experience an accelerated decline in eGFR over time.^[11]^ However, the incidence of, and temporal relationship between, adverse events and anticoagulation strategies in patients with HF, AF, and ESKD are not well documented.

We hypothesized that real-world data should be analyzed to evaluate the incidence of bleeding and thromboembolic events in patients with HF and ESKD, with the aim of clarifying patterns of apixaban prescription for AF. These findings could guide apixaban dose selection, improve prognosis, and inform clinical trial design to better include patients with severe kidney dysfunction, thereby enhancing the applicability of research findings.

## 2. Methods

### 2.1. Participants

We recruited 825 patients (mean age: 77.9 ± 13.4 years) with ESKD who were undergoing routine follow-up at a nephrology outpatient clinic and a hemodialysis center. Participants were enrolled between October 2018 and December 2024. All patients were screened for medications and medical conditions.

**Figure 1.**
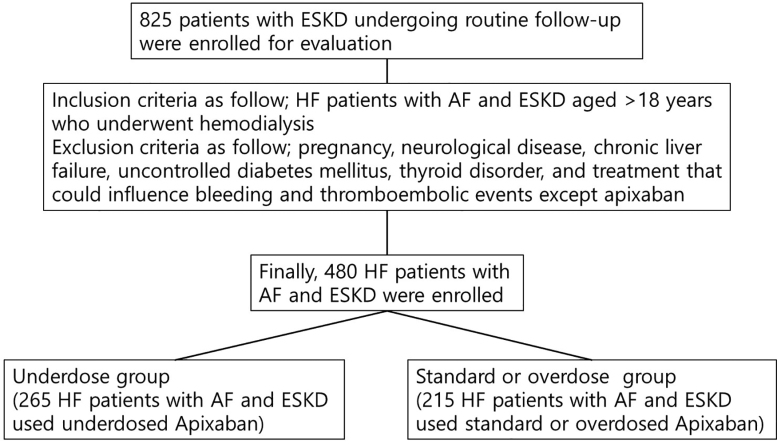
Study flow and schematics.

The study population comprised patients aged > 18 years with HF, AF, and ESKD, with or without HD. The main exclusion criteria were pregnancy, neurological disease, chronic liver failure, uncontrolled diabetes mellitus (DM), thyroid disorders, and treatments that could influence bleeding or thromboembolic events, with the exception of apixaban. Ultimately, 480 patients with HF, AF, and ESKD (245 males, 235 females; mean age: 77.3 ± 10.1 years) who completed clinical follow-up were included in the analysis (Fig. 1). HF was diagnosed according to the 2022 guidelines of the European Society of Cardiology.^[12]^ Patients were classified as having HF with reduced ejection fraction if they had a left ventricular ejection fraction (LVEF) < 50%, symptoms or signs of HF, N-terminal pro-B-type natriuretic peptide (NT-proBNP) levels > 125 ng/L, and evidence of relevant structural heart disease.^[13]^ All patients were followed by providers from the cardiology and nephrology departments. All relevant data are included in the paper and its Supporting Information files. In this study, we analyzed patients with HF, AF, and ESKD according to off-label apixaban use in real-world practice at our center in Korea.

### 2.2. Definitions of Apixaban dose (standard, underdose, and overdose)

The criteria for apixaban dose reduction based on reduced creatinine clearance required the presence of at least two of the following: age ≥ 80 years, body weight ≤ 60 kg, and serum creatinine level ≥ 1.5 mg/dL.^[14]^ The standard apixaban dose was defined as the dose prescribed according to these guideline recommendations; underdose and overdose were defined as doses lower or higher, respectively, than those recommended by the guidelines.

### 2.3. Ethical statement

The study protocol and the requirement for informed consent from individual patients were approved by the Ethics Committee of Kosin University Gospel Hospital (IRB No. 2022-06-012). Written informed consent was obtained from all patients. The study was conducted in accordance with the principles of the 2013 Declaration of Helsinki.

### 2.4. Study endpoints

The primary outcomes were major bleeding, minor bleeding, and clinically relevant nonmajor bleeding, as defined by the International Society on Thrombosis and Hemostasis (ISTH) criteria, according to apixaban dose in patients with HF, AF, and ESKD.^[15]^ In studies in which ISTH criteria were not explicitly stated, these criteria were applied if sufficient data were available. The primary efficacy outcome was treatment failure, defined according to the study context as recurrence of venous thromboembolism (VTE) and arterial thromboembolism (ATE) (stroke, myocardial infarction, and cardioembolic events).

Secondary outcomes included independent parameters predictive of bleeding and thromboembolic events, including stroke, according to apixaban dose in patients with HF, AF, and ESKD.

### 2.5. Outcome definitions

Major bleeding was defined according to the ISTH criteria as fatal bleeding, symptomatic bleeding in a critical area or organ, or bleeding causing a decrease in hemoglobin of 20 g/L or requiring transfusion of two or more units of whole blood or red blood cells.^[15]^ VTE recurrence was defined as objectively confirmed fatal or nonfatal deep vein thrombosis of the leg or pelvis, pulmonary embolism, or death to which pulmonary embolism contributed or could not be ruled out.^[16]^ ATE events included stroke, myocardial infarction, or cardioembolic events, such as acute limb ischemia. Minor bleeding and clinically relevant nonmajor bleeding were defined according to ISTH criteria.^[17]^

### 2.6. Data collection

After electrocardiography (ECG) and chest radiography were performed, each patient’s cardiovascular status was evaluated using echocardiography and blood laboratory data obtained at the initial visit, as determined by the attending physicians. The following information was collected from the database: sex, age, height, and weight; cardiovascular risk factors, including hypertension (use of antihypertensive agents, systolic blood pressure ≥ 140 mm Hg, or diastolic blood pressure ≥ 90 mm Hg on admission) and DM; use of oral hypoglycemic agents or insulin, or glycosylated hemoglobin levels ≥ 6.5%); cardiovascular disease status, including structural heart disease, congestive HF, or a history of disabling cerebral infarction or transient ischemic attack; and medication use.

### 2.7. ECG-monitoring device to detect AF

The HiCardi (Mezoo Co., Ltd., Wonju-si, Gangwon-do, Korea) is an 8 g wearable ECG-monitoring patch device measuring 42 × 30 × 7 mm (without disposable electrodes) that is certified as a medical device by the Ministry of Food and Drug Safety of Korea. This device monitors and records single-lead ECG, respiration, skin-surface temperature, and activity data. The ECG signal was recorded at a sampling frequency of 250 Hz with 14-bit resolution. Data from the wearable patches were transferred via Bluetooth Low Energy to a mobile gateway implemented as a smartphone application, which then transmitted the data to a cloud-based monitoring server. After informed consent was obtained, the wearable patch was attached to the left sternal border. ECG signals and the aforementioned data were continuously recorded, and all ECG signals were reviewed by a cardiologist using the cloud-based monitoring server.

### 2.8. Statistical analysis

All continuous variables are expressed as the mean ± standard deviation or median (25th and 75th interquartile range), depending on their distribution. The statistical significance of differences in continuous variables was assessed using the Student *t* test or the Mann–Whitney *U* test, as appropriate. Categorical variables are presented as frequencies (percentages) and were analyzed using the chi-square test. To identify variables independently associated with clinical outcomes according to apixaban dose, multivariate analysis was performed using logistic regression for variables with *P* values < .05 in univariate analysis. Correlations were assessed using Spearman rank correlation test. All statistical analyses were conducted using SPSS statistical software (version 19.0; SPSS Inc., Chicago), and statistical significance was set at *P* < .05 (2-sided).

## 3. Results

Among 480 patients with HF, AF, and ESKD (age: 77.3 ± 10.1 years), 265 (55.2%) received off-label underdoses of apixaban. Baseline characteristics and echocardiographic parameters are shown in Table 1. No significant differences in baseline characteristics, including CHA_2_DS_2_-VASc and HAS-BLED scores, were observed among the groups.

**Table 1:** Baseline demographic and echocardiographic parameters in heart failure patients with atrial fibrillation and end stage kidney disease.

Variables	Underdose group (n = 265)	Standard or overdose group (n = 215)	*P*-value
Age (years)	76.1 ± 9.9	78.6 ± 10.3	.890
Sex (male, %)	136 (51.3)	109 (50.7)	.927
DM (%)	154 (58.1)	119 (55.3)	.579
HTN (%)	248 (93.6)	199 (92.6)	.718
CAD (%)	183 (69.1)	140 (65.1)	.380
CVA (%)	36 (13.6)	29 (13.6)	1.000
HF (%)	38 (11.3)	35 (11.8)	.386
CHA2DS2-VASc	2.5 ± 1.7	2.7 ± 0.7	.188
HAS-BLED	2.6 ± 0.8	2.7 ± 0.8	.102
Hemodialysis (%)	3 (1.1)	3 (1.3)	.349
Medications			
Apixaban dose (mg)	5.1 ± 1.4	6.6 ± 2.7	<.001
Beta-blockers (%)	183 (69.1)	152 (70.7)	.611
CCB (%)	71 (26.8)	65 (30.2)	.351
ACEi or ARB (%)	104 (39.2)	69 (32.1)	.126
Antiarrhythmics (%)	120 (45.3)	90 (41.9)	.461
Diuretics (%)	121 (45.7)	112 (52.1)	.169

HF = heart failure; AF = atrial fibrillation; ESKD = end stage kidney disease; DM = diabetes mellitus; HTN = hypertension; CAD = coronary artery disease; CVA = cerebrovascular accident; CHA_2_DS_2_-VASc score = a point-based system that estimates the risk of stroke in patients with AF [calculated based on the presence of HF (1 point), HTN (1 point), age ≥ 75 years (2 points), age 65–74 years (1 point), DM (1 point), previous stroke or TIA (2 points), female sex (1 point), and vascular disease (1point)]; HAS-BLED score, risk score for predicting bleeding risk in anticoagulated patients with AF [HTN, abnormal renal/liver function, stroke, bleeding history or predisposition, labile INR, elderly, and drugs/alcohol concomitantly]; CCB = calcium channel blocker; ACEi = angiotensin-converting enzyme inhibitor; ARB = angiotensin receptor blocker.

Echocardiographic and laboratory findings are presented in Table 2, with no significant differences observed between groups.

**Table 2:** Echocardiographic and laboratory findings in heart failure patients with atrial fibrillation and end stage kidney disease.

Variables	Underdose group (n = 265)	Standard or overdose group (n = 215)	*P*-value
Echo parameters			
LVEF (%)	42.1 ± 7.8	42.2 ± 7.4	.862
LVIDs (mm)	29.9 ± 5.0	30.3 ± 6.4	.748
LVIDd (mm)	45.5 ± 5.2	45.7 ± 6.3	.826
IVSD (mm)	11.1 ± 1.9	10.8 ± 1.6	.381
LVPWD (mm)	10.5 ± 5.3	10.1 ± 4.8	.586
LAD (mm)	36.2 ± 7.4	35.9 ± 6.1	.824
E velocity (cm/sec)	0.6 ± 0.2	0.6 ± 0.2	.824
E/E’	12.8 ± 3.8	12.9 ± 6.5	.623
Laboratory findings			
BUN (mg/dL)	31.3 ± 16.3	31.7 ± 15.4	.762
Creatine (mg/dL)	2.5 ± 0.5	2.3 ± 0.4	.269
BNP (pg/dL)	1098.1 ± 23.4	1102.1 ± 20.1	.980
Na (mEq/L)	137.7 ± 3.8	137.3 ± 4.2	.373
K (mEq/L)	4.4 ± 0.7	4.4 ± 0.6	.562
Cl (mEq/L)	104.2 ± 5.2	104.4 ± 4.8	.662
CO_2_ (mEq/L)	24.2 ± 4.1	23.7 ± 4.3	.271
Hb (g/dL)	11.4 ± 1.9	11.4 ± 2.0	.872
Platelet *10^3^/uL	182.3 ± 70.3	189.2 ± 81.1	.325

AF = atrial fibrillation, BNP = B-type Natriuretic Peptide, BUN = blood urea nitrogen, E = peak mitral flow velocity of the early rapid filling wave, E’ = early diastolic mitral annulus velocity, ESKD = end stage kidney disease, Hb = hemoglobin, HF = heart failure, IVSD = interventricular septal diameter, LAD = left atrial diameter, LVEF = left ventricular ejection fraction, LVIDd = left ventricular diastolic diameter, LVIDs = left ventricular systolic diameter, LVPWD = left ventricular posterior wall diameter.

Univariate analysis demonstrated that the CHA_2_DS_2_-VASc score was associated with stroke and systemic embolic events according to off-label apixaban dose in patients with HF, AF, and ESKD (Table 3A). Similarly, DM and HAS-BLED scores were associated with total bleeding events in the univariate analysis. In multivariate analysis, the HAS-BLED score remained an independent predictor of total bleeding events in patients with HF, AF, and ESKD (Table 3B).

**Table 3:** Univariate and multivariate Cox analyses for (A) stroke/systemic embolism events and (B) total bleeding according to the off-label dose of apixaban in heart failure patients with atrial fibrillation and end stage kidney disease.

(A)	Univariate analysis		Multivariate analysis	
Variable. N (%)	OR (95% CI)	*P*-value	OR (95% CI)	*P*-value
CHA_2_DS_2_-VASc score	1.818 (1.081–3.058)	0.024	1.818 (1.081–3.058)	.024
(B)	Univariate analysis		Multivariate analysis	
Variable. N (%)	OR (95% CI)	*P*-value	OR (95% CI)	*P*-value
DM (%)	11.769 (1.401–98.853)	0.023		
HAS-BLED score	2.944 (1.318–6.578)	0.008	2.614 (1.098–6.224)	.030

CI = confidence interval, OR = odds ratio; CHA2DS2-VASc score, a point-based system that estimates the risk of stroke in patients with atrial fibrillation [calculated based on the presence of heart failure (1 point), hypertension (1 point), age ≥ 75 years (2 points), age 65–74 years (1 point), DM (1 point), previous stroke or TIA (2 points), female sex (1 point), and vascular disease (1point)]; DM = diabetes mellitus; HAS-BLED score, risk score for predicting bleeding risk in anticoagulated patients with atrial fibrillation [hypertension, abnormal renal/liver function, stroke, bleeding history or predisposition, labile INR, elderly, and drugs/alcohol concomitantly].

**Figure 2.**
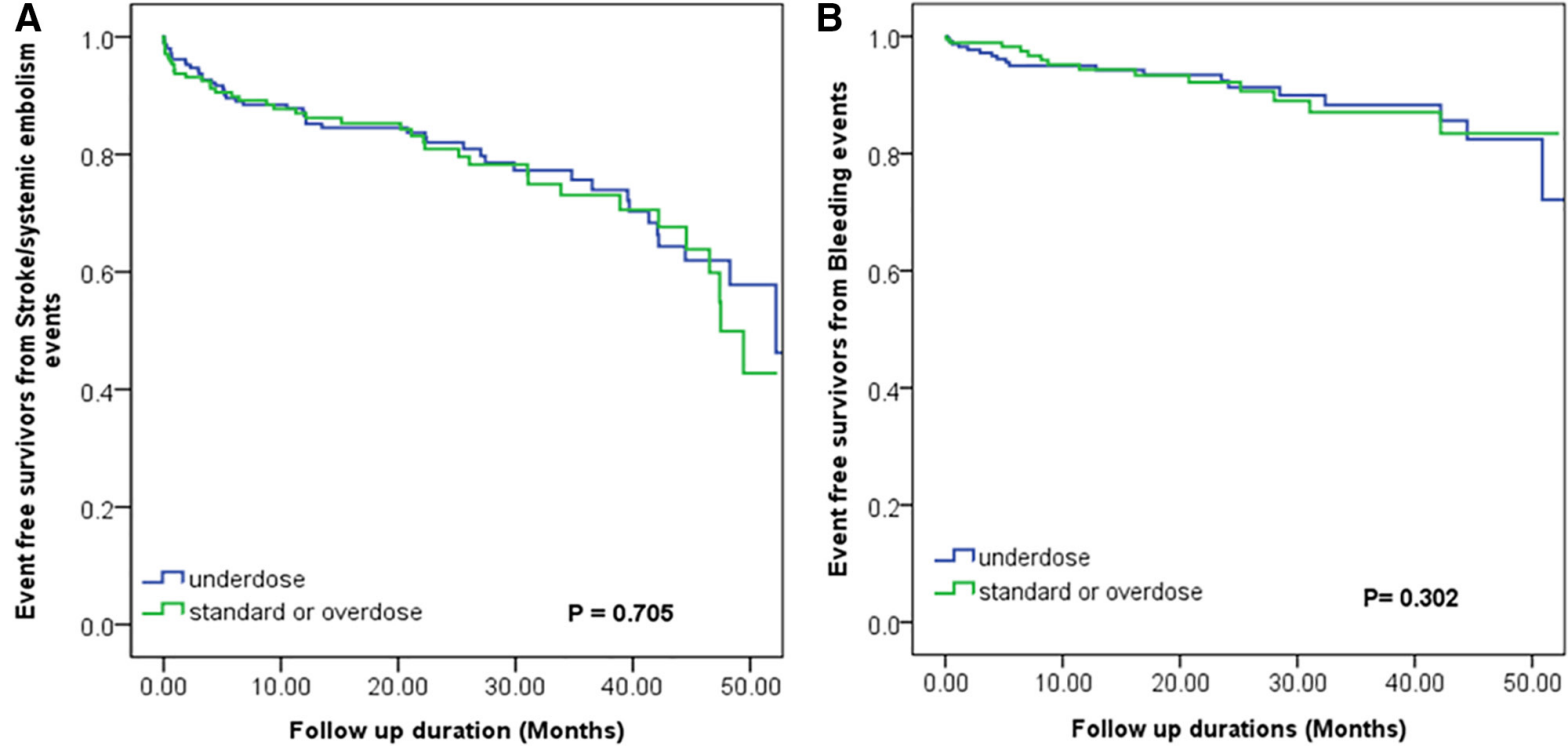
Kaplan–Meier curve for (A) stroke/systemic embolism events and (B) total bleeding according to the off-label dose of apixaban in heart failure patients with atrial fibrillation and end stage kidney disease.

During a median follow-up of 48 months, no significant differences were observed in systemic thrombotic events, including stroke and systemic embolism, or in all-cause mortality between the off-label underdose group and the standard-dose or off-label overdose groups (*P* = .705; Fig. 2A). In addition, no significant differences in bleeding events were observed among the different apixaban dose groups (*P* = .302; Fig. 2B) (off-label underdose vs standard dose, *P* = .600; off-label overdose vs standard dose, *P* = .395; off-label underdose vs off-label overdose, *P* = .469). However, in multivariate analysis, the CHA_2_DS_2_-VASc score was an independent risk factor for systemic thrombotic events (odds ratio [OR]: 1.818, confidence interval [CI]: 1.081–3.058; *P* = .024).

The incidence of bleeding events did not differ significantly between groups (underdose group vs standard or overdose groups: 6.55 vs 8.03 per 100 person-years, *P* = .354). Similarly, no significant difference in thromboembolic event rates was observed between groups (underdose group vs standard or overdose groups: 17.58 vs 18.01 per 100 person-years, *P* = .132).

## 4. Discussion

In this study, we evaluated the effects of different real-world, off-label apixaban dosing regimens on long-term clinical outcomes in patients with HF, AF, and ESKD. Overall, apixaban use was associated with favorable outcomes across all patient groups, with acceptable safety profiles.

Previous studies have demonstrated an association between CKD and the prevalence of HF, including both HF with reduced ejection fraction and HF with preserved ejection fraction.^[18]^ Furthermore, data from community-dwelling older adults in the Cardiovascular Health Study showed that CKD with an eGFR < 45 mL/min per1.73 m^2^, as estimated by the Chronic Kidney Disease Epidemiology Collaboration (CKD-EPI) equation, was associated with incident HF.^[19]^ In this population, arrhythmias such as AF during HD are common, and dialysis-related electrocardiographic artifacts mimicking arrhythmias have also been reported.^[20]^ Many patients with HF experience an accelerated decline in eGFR over time. In real world practices, acute kidney injury on ESKD commonly occur during the end stage HF, particularly those with lower baseline eGFR, with higher incidence preceding death. Greater awareness of kidney-related complications and further research are essential to improve clinical prognosis in those patients.^[10]^ However, the incidence of, and temporal relationship between, adverse kidney events and arrhythmias, including AF-related mortality and events such as stroke, in patients with HF remain poorly characterized.

Overall, clinical outcomes were similar between patients who received appropriate and potentially inappropriate dose adjustments of direct oral anticoagulants (DOACs). However, a previous study^[21]^ reported that, among patients with AF and renal dysfunction, defined as an estimated creatinine clearance < 60 mL/min, those who were potentially undertreated experienced a statistically significant increase in major adverse cardiovascular and neurological events compared with patients who received appropriate treatment based on the modification of diet in renal disease formula.

When the impact of DOACs on safety outcomes was evaluated by classifying them according to the indication for anticoagulant administration, DOAC use was found to be associated with a reduction in major bleeding events in patients with stage 5 chronic kidney disease or those receiving renal replacement therapy. DOACs have been shown to be associated with a reduction in major bleeding events in patients with AF as well as in patients with stage 4 and unspecified chronic kidney disease.^[22]^

Current guidelines indicate that apixaban and rivaroxaban are safe for patients with stage 4 CKD.^[23,24]^ Previous studies also support the use of DOACs (with the exception of dabigatran, in patients with stage 4 CKD. Although DOACs remain contraindicated in patients receiving dialysis under European and Canadian guidelines (European Heart Rhythm Association, European Society of Cardiology, Canadian Cardiovascular Society, and Thrombosis Canada), apixaban may be considered in patients with severe renal disease or those undergoing dialysis based on the US Food and Drug Administration (FDA)–approved package insert and the guidelines of the American Heart Association and the American College of Chest Physicians.^[23,25,26]^ Current FDA product information approves the use of rivaroxaban in patients with an eGFR of <15 mL/min, but other guidelines do not recommend its use in this patient group.^[22,27]^ These findings are plausible, as the safety of apixaban and rivaroxaban would theoretically be least affected by impaired renal function and they have the lowest potential for inappropriate drug accumulation based on DOAC pharmacological profiles.

To the best of our knowledge, this is the first study to demonstrate the impact of real-world, off-label apixaban dosing on clinical outcomes in patients with HF, AF, and ESKD. There are no clinical outcome data on the use of DOACs in patients with HF and a creatinine clearance of 30 mL/min, as calculated using the Cockcroft–Gault equation, including those undergoing HD, for whom warfarin provides uncertain benefits.^[28]^ Until data from randomized clinical trials become available, warfarin remains the preferred anticoagulant for these patient subgroups.^[27]^ Furthermore, the FDA has approved apixaban for patients undergoing HD without safety data from this population.^[14]^ Therefore, in this study, we evaluated the safety profile of off-label apixaban dosing in patients with HF, AF, and ESKD and found no significant differences in safety outcomes, including bleeding and systemic thromboembolic events, between dose groups.

Our study has several limitations that warrant consideration. First, the relatively small sample size limits the generalizability of the findings to patients with HF, AF, and ESKD undergoing HD. However, the sample size was sufficient to identify significant correlations between stroke, systemic embolism, or bleeding events and off-label apixaban dosing. Despite the limited number of patients, our analysis demonstrated no significant differences in long-term clinical outcomes, particularly among patients with HF. Therefore, these findings should be considered hypothesis-generating, and future prospective studies are needed to confirm the results. Second, there is a potential risk of model overfitting, given the number of adjustment covariates relative to the number of events for some outcomes. In addition, the mean follow-up period for the assessment of clinical outcomes was 5 years, which further limited the number of observed clinical events. Third, data were unavailable for several important clinical parameters, including hemodialysis-related variables, degree of anuria, blood pressure, N-terminal pro-B-type natriuretic peptide levels, albuminuria, cystatin C levels, cardiac index, pulmonary capillary wedge pressure, and the etiology of kidney dysfunction (e.g., exposure to nephrotoxins such as contrast agents, nonsteroidal anti-inflammatory drugs, or hemodynamic deterioration). The absence of these data may have resulted in residual and unmeasured confounding, potentially affecting the strength of the observed associations. In addition, the impact of medication changes on eGFR during follow-up was not analyzed, representing another limitation. Although we reported the distribution of HF therapies across ESKD groups, this study did not assess the causal effects of these therapies on outcomes. Furthermore, HF management was likely not evenly distributed across ESKD groups, as patients with more advanced ESKD may face greater challenges in receiving certain treatments, such as angiotensin converting enzyme inhibitors, angiotensin receptor blockers, or angiotensin receptor-neprilysin inhibitors, owing to contraindications or perceived risks. These disparities could have contributed to differences in outcomes and should be explored in future studies. Despite these limitations, this study provides valuable insights into adverse clinical events in patients with HF, AF, and ESKD and serves as a foundation for further research and clinical decision-making in this important area.

In conclusion, no significant differences were observed in systemic thrombotic events, including stroke or systemic embolism, mortality, or bleeding risk among patients with HF, AF, and ESKD, supporting apixaban dosing tailored to individual clinical characteristics in real-world practice.

## Acknowledgements

We thank all members of division of cardiology and nephrology for providing data and technical support, and for providing follow-up information.

## Author contributions

**Conceptualization:** Sung Il Im.

**Data curation:** Sung Il Im, Su Hyun Bae.

**Formal analysis:** Sung Il Im, Soo Jin Kim.

**Funding acquisition:** Bong Joon Kim.

**Investigation:** Jung Ho Heo.

**Methodology:** Ye Na Kim.

**Project administration:** Yeonsoon Jung.

**Resources:** Hark Rim.

**Software:** Sung Pil Cho.

**Supervision:** Sung Il Im, Jung Hwan Park.

**Validation:** Ho Sik Shin.

**Visualization:** Ho Sik Shin.

**Writing – original draft:** Sung Il Im.

**Writing – review & editing:** Sung Il Im, Ho Sik Shin.
